# A Biologically Inspired Approach for Robot Depth Estimation

**DOI:** 10.1155/2018/9179462

**Published:** 2018-08-23

**Authors:** Ester Martinez-Martin, Angel P. del Pobil

**Affiliations:** ^1^RoViT, University of Alicante, 03690 Sant Vicent del Raspeig, Spain; ^2^RobInLab, Universitat Jaume-I, 12071 Castellón, Spain; ^3^Sungkyunkwan University, Seoul, Republic of Korea

## Abstract

Aimed at building autonomous service robots, reasoning, perception, and action should be properly integrated. In this paper, the depth cue has been analysed as an early stage given its importance for robotic tasks. So, from neuroscience findings, a hierarchical four-level *dorsal* architecture has been designed and implemented. Mainly, from a stereo image pair, a set of complex Gabor filters is applied for estimating an egocentric quantitative disparity map. This map leads to a quantitative depth scene representation that provides the raw input for a qualitative approach. So, the reasoning method infers the data required to make the right decision at any time. As it will be shown, the experimental results highlight the robust performance of the biologically inspired approach presented in this paper.

## 1. Introduction

Robotics research in industrial settings has resulted in a productivity improvement and a reduction in danger for humans. From this success, the Robotics community is aiming to build autonomous robot systems able to assist human beings in any activity (from their mundane tasks (e.g., cooking or cleaning) to more intellectual and social efforts of entertainment and caregiving). Along these lines, all aspects of intelligent processing—from perception to action—must be engaged and integrated. That is, the developed robots should be capable of completing their tasks by properly interacting with their environment, even when it is dynamic and unknown. Nevertheless, most of the current robot systems compute their actions from their perceived input by using models of the environment and, consequently, they cannot imagine other models when they find themselves in unforeseen situations. Therefore, a key point in any autonomous system is its adaptability to the environment and its changes.

From a biological point of view, humans use many kinds of sensory information (i.e., sight, hearing, smell, taste, and/or touch) to obtain a complete and reliable representation of their surroundings. In this sense, though the understanding of the human brain still remains a great challenge [1], neuroscience research is making progress towards understanding how human neurons encode information and how the complex dynamical interactions within and among neuronal networks lead to learning and produce sensorimotor coordination and motor control [2]. Apparently, the strategy adopted by all the superior vertebrates consists of separating the recognition of an object (the *what* problem) from finding its position (the *where* problem) [3]. Thus, the temporal regions of the cerebral cortex are involved in the *what* pathway, while the parietal regions try to find *where* the target objects are [4–6]. Actually, a wide range of neurobiological studies have analysed the organization and connectivity of sensory and motor areas in the mammalian cerebral cortex. In particular, many researchers have focused on the visual cortex, given its importance (around 55% of the neocortex of the primate brain is concerned with vision [3]).

On this topic, cognitive studies show that the knowledge about the retinoptic image of a visual object is qualitative, although this image is quantitative by nature (i.e., the retina locations are stimulated by light of a specific spectrum of wavelengths and intensity [7]). So, the qualitative sensory data allows the system to abstract the relevant features of the environment and to extract more information by means of qualitative reasoning processes, otherwise unavailable.

According to the neuropsychological research [8–13], mental processes form a hierarchy of mental representations with maximally egocentric representations at the bottom and maximally allocentric representations at the top, progressively abstracting away from the particularities of the egocentric representations. So, the processing pipeline of the primate brain is hierarchically organised into 8 to 10 levels such that the neurons in the early visual areas extract simple image features (e.g., motion, orientation, colour, etc.) over small local regions of visual space and this quantitative information is transmitted to neurons in higher visual areas responding to even more complex features and covering larger regions of the visual field [14]. That is, the neuronal visual processing starts in the retina of the left and right eyes. Then, the low-level processing is performed by the cortical areas V1 and V2, providing input for both the ventral (*what*) and the dorsal (*where*) streams, which perform a deeper analysis. So, as the ventral pathway is involved in object recognition and categorization, the complexity of the extracted features increases up to an object level for specific object classes like faces [15]. On its behalf, the dorsal pathway is engaged in the analysis of space and in action planning and, similarly to the ventral stream, the complexity of the stimulus features increases progressively such that the higher areas encode the location of stimuli in spatial or head-fixed coordinates.

### 1.1. Contributions

In this paper, we have simplified the mammalian brain model by focusing on depth estimation when a visual input is considered. Given that the dorsal *where* pathway deals with localization of scene objects, a four-level *dorsal* architecture for depth estimation is presented. In rough outlines, from a stereo image pair (an *egocentric* representation of the scene in image coordinates), the system quantitatively estimates an *egocentric* depth map of the observed scene. From that information, *egocentric* qualitative depth information is generated to be able to infer the corresponding *allocentric* depth map. Note that this *allocentric* data can easily be transformed to the necessary *egocentric* motor coordinates to properly perform the task at hand. Thus, this *egocentric-allocentric* combination provides the system with the vision-for-action and vision-for-perception models, by improving the efficiency in the coordination of sensory input and motor output.

In addition, this *egocentric-allocentric* integration allows the system to locate objects in the scene along time in a more precise way by properly dealing with the lack of information and/or data uncertainty. That is, according to biological findings, the *egocentric* representation is temporary and it depends on the robot's location. On the contrary, the *allocentric* representation is independent on the robot's situation and, in addition, it survives over extended periods of time not relying on immediate visual input. Therefore, *allocentric* data provides the system with object's spatial information in the robot's surroundings, even when those objects are not visible. So, the robot system could always plan a path free of obstacles (visible or not) to manipulate an object at any time.

With that purpose, this paper is organised as follows: a brief overview of the system is given in Section 2.  Section 3 introduces our algorithm for 2D binocular depth estimation based on phase difference in the Fourier domain. Its output is the input to a qualitative spatial inference mechanism, introduced in Section 4. Some experimental results are presented in Section 5 and discussed in Section 6.

## 2. System Overview

One of the great research challenges is to build autonomous systems completely based on biological models. In this sense, general vision problems are divided into more specific tasks in order to be biologically solved such as simultaneous localization and mapping (SLAM) [16, 17], navigation [18, 19], or feature extraction [20, 21]. Nevertheless, these approaches present *flat processing schemes* unlike the hierarchical structure of the biological systems.

**Figure 1:**
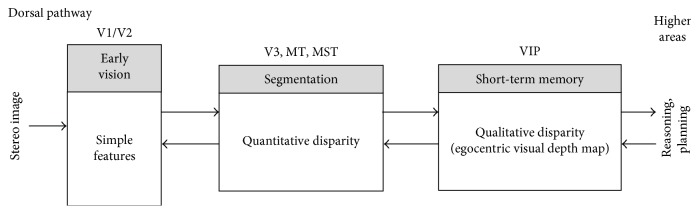
Overview of our biologically inspired active vision system for depth.

Going a step further, we have designed and implemented a four-level architecture based on the dorsal pathway with the purpose of providing robotic systems with biological depth estimation.  Figure 1 gives an overview of our system that can be summarised as follows: the early vision area (V1/V2) is modelled as a set of complex Gabor filters with a cosine-based real part and a sine-based imaginary part. This processing at multiple scales and orientations, results in a quantitative stereo disparity estimation that leads to a quantitative depth map. From this knowledge, an egocentric representation of the object localization within the scene is obtained by following a qualitative approach. Finally, a qualitative reasoning method allows the system to infer new information and make decisions more accurately.

### 2.1. Depth Estimation: A Quantitative Approach

The first issue to be solved is how to perceive depth (i.e., the distance of individual points from the observer) from 2D visual fragments. This is a classic problem in the field, analysing different visual cues based on the *raw* data about the 3D layout of objects they provide.

In this context, one of the major categories for depth perception is *visual monocular cues* such as shadows, motion parallax, linear perspective, or occlusion. Nevertheless, these cues provide *relative* distance considering that they provide information about the object's location (or its parts) with respect to other objects in the scene (i.e., *allocentric* representation). This representation is independent of the system's current location since it is indexed to a world-based coordinate system, and it survives over extended periods of time [8]. Note that this representation must be built up from vision over time, but it does not rely on immediate visual input.

As a solution, binocular disparity can be used since it provides *absolute* distances by triangulating the distance to an object. Thus, this cue provides an *egocentric* representation, which is temporary and is based on the object's directions relative to the current system's pose within the scene. Consequently, the depth encoder will allow the system to act upon its environment with the aim of locating, reaching, and/or manipulating objects.

Focusing on obtaining a *disparity map* and given that two images from different points of view are required, the first issue to be solved is the correspondence problem. Basically, it refers to the problem of matching *corresponding* image points in the stereo pair images. Although a large number of algorithms have been proposed, they can be classified into two main groups [22]:

- *Feature-based* matching algorithms. They concern two steps:
  - *Feature extraction.* Features such as edges, colour, and so on are extracted from the images and used as key points for the correspondence method.
  - *Solving the correspondence problem*. A correspondence between image elements is chosen from among the many conceivable ones. Various types of knowledge, constraints, and plausibility considerations are used at this stage:
    1. Search space: for an element in the left image, a matching element is sought only within a certain region of the right image.
    2. Feature attributes: in the case the image elements can be distinguished from one another, then only those of the same type (line terminations, edges) and with the same characteristics (e.g., polarity of contrast, colour) are matched.
    3. Ordering constraints: because the plausibility of other matches changes once a match between two features has been established.

Note that this method results in sparse disparity maps since it only gets disparities for the extracted features.

(ii) *Area-based* matching algorithms. They try to match each pixel independently of the image content. Therefore, the resulting disparity map can be very dense what makes them be an interesting alternative to quantify and solve early vision problems.

As our goal is to extract dense and reliable depth information from the observed scene, stereo-matching algorithms based on area have been studied. Nevertheless, matching correspondence approaches cannot be efficiently adapted to changing camera geometry information, a very common situation when a robot system performs its activities in real scenarios. Consequently, nearly all the proposed stereo vision methods divide their performance into two stages: camera calibration (used for stereo rectification) and dense disparity estimation. With regard to the calibration step, it is typically performed offline by means of feature-based methods [23–25]. Note that this stage is problematic when a noisy visual input is processed and/or the stereo system is decalibrated.

As an alternative, biological studies have revealed that the response of visual cortex is turned to the band-limited portion of the frequency domain. That is, the brain decomposes the spectra into perceptual channels that are bands in spatial frequency [26]. Similarly, images can be seen as sinusoidal functions moved in depth such that the same gray value function appears in both images of a stereo pair at different phase angle. This fact leads to the possibility of extracting disparity by using frequency filters. In this context, Gabor functions have been extensively used because of their similarity with the receptive field of cells in the virtual cortex (e.g., [27–29]). In fact, they have been particularly successful in many computer vision and image-processing applications [30, 31]. However, a fundamental problem with these approaches is the inherently large memory and computational overheads required for training and testing in the overcomplete Gabor domain, what makes them hardly suitable for real-time robot applications.

As a solution, we propose an approach for *disparity* estimation based on phase-difference that does not require precise calibration information (in terms of the relative transformation (position and orientation) between the two cameras) that provides accurate results in real time. Thus, given that the presented approach does not use the external camera parameters, cameras are only calibrated at the beginning of the experiment to obtain internal camera parameters, and no more calibration procedure is performed although the system's location has changed. In addition, this approach is robust to changes in contrast, scale, and orientation, what is very important in the context of disparity estimation.

Mainly, the *phase-based binocular disparity estimation* (PBBDE) approach (see [32] for a detailed description) assumes that an image is a sinusoidal gray value function moved in depth such that the difference in the object's position in the stereo input (i.e., *disparity*) is estimated as the phase difference between the two images. For that, and considering the *x-y* image dimensionality, a bank of 2D-oriented Gabor filters is used. The different orientations, *θ*_*q*_, are evenly distributed and equal to (*qπ*)/*K*. Let *q* be in the range from 0 to *K* − 1, while *K*=8 orientations are considered in our implementation. Thus, for a specific orientation *θ*_*q*_, the spatial phase at pixel location **x**=(*x*, *y*)^*T*^ is extracted as follows:

(1) $$\begin{array}{c} Q_{q}\left(x\right)=\left(I\;*\;f_{q}\right)\left(x\right)=\rho_{q}\left(x\right)e^{j\varphi_{q}}=C_{q}\left(x\right)+jS_{q}\left(x\right), \end{array}$$

where $\rho_{q}\left(x\right)=\sqrt{C_{q}{\left(x\right)}^{2}+S_{q}{\left(x\right)}^{2}}$ and *φ*_*q*_(**x**)=arctan(*S*_*q*_(**x**), *C*_*q*_(**x**)) are the amplitude and the phase components, respectively, and *C*_*q*_(**x**) and *S*_*q*_(**x**) are the responses of the quadrature filter pair. The *∗* operator corresponds to the convolution operation.

In this way, the *1D disparity* can be estimated from each oriented filter response (at orientation *θ*_*q*_) by projecting the phase difference along the direction of the epipolar lines as

(2) $$\begin{array}{c} \delta \left(x\right)=\frac{{\left[\varphi^{L}\left(x\right)-\varphi^{R}\left(x\right)\right]}_{2\pi }}{\omega \left(x\right)}=\frac{{\left[\Delta \varphi \left(x\right)\right]}_{2\pi }}{\omega \left(x\right)}, \end{array}$$

where *φ*^*L*^(**x**) and *φ*^*R*^(**x**) represent the phase values of band-pass-filtered versions and *ω*(**x**) is the average instantaneous frequency of the band-pass signal at point **x** and, under a linear phase model, it can be approximated by *ω*_0_(**x**) [33]. Nevertheless, with the aim of generalizing the approach for active stereo vision systems, the 2D disparity (*δ*(**x**)) is obtained by differencing the combination of disparity estimates (*δ*_*c*,*θ*_) of the bank filters as follows:

(3) $$\begin{array}{c} \delta^{*}\left(x\right)=\left[\underset{\theta }{\sum }d_{x,\theta }\;\underset{\theta }{\sum }d_{y,\theta }\right]\left[\begin{array}{cc} \sum_{\theta }\frac{d_{x,\theta }\;d_{x,\theta }}{d_{x,\theta }^{2}+d_{y,\theta }^{2}} & \sum_{\theta }\frac{d_{x,\theta }\;d_{y,\theta }}{d_{x,\theta }^{2}+d_{y,\theta }^{2}}\\ \sum_{\theta }\frac{d_{y,\theta }\;d_{x,\theta }}{d_{x,\theta }^{2}+d_{y,\theta }^{2}} & \sum_{\theta }\frac{d_{y,\theta }\;d_{y,\theta }}{d_{x,\theta }^{2}+d_{y,\theta }^{2}} \end{array}\right], \end{array}$$

where *d*_*x*,*θ*_ and *d*_*y*,*θ*_ are the projection of *δ*_*c*,*θ*_ along the horizontal and vertical axis, respectively. In this way, multiple disparity estimates are obtained at each location. These estimates can be integrated over the different pyramid levels. For that a disparity map is first computed at the coarsest level. Then, this disparity estimation is up sampled by means of an expansion operator and a method to double, in order to make it compatible with the next level estimation. After that sample up, the obtained map is used to reduce the disparity at level *n*+1, by warping the right filter responses before computing the phase difference:

(4) $$\begin{array}{c} \delta^{n}=\frac{{\left[\varphi^{L}\left(x^{\prime }\right)-\varphi^{R}\left(x^{\prime }\right)\right]}_{2\pi }}{k\left(x\right)}\;+\;\left(2\;\;\mathrm{expand}\left(\delta^{n-1}\right)\right), \end{array}$$

where **x**′=(**x**+*d*_*x*_^*n*−1^(**x**), *y*+*d*_*y*_^*n*−1^(**x**)), being *d*_*x*_^*n*−1^ the horizontal disparity at the level *n* − 1 and *d*_*y*_^*n*−1^ the vertical disparity at the level *n* − 1. Consequently, the remaining disparity is guaranteed to lie within the filter range. This procedure is repeated until the finest level is reached.

Once the disparity map has been generated, the following step is to infer the object's depth within the visual scene. For that and considering that a convergent stereo camera system could be used, the depth estimation is obtained by

(5) $$\begin{array}{c} z=f\frac{4+{\left(b/f\right)}^{2}}{2\left(\left(b/f\right)-2\;\;\tan\left(\delta /2\right)\right)}\tan\left(\frac{\delta }{2}\right), \end{array}$$

where *b* represents the intercamera distance, *δ* corresponds to the binocular disparity, and *f* is the focal length. Note that the binocular disparity is expressed in radians in this formula. Therefore, the camera-centred polar coordinates are considered instead of the Cartesian ones.

In this way, disparity and depth are properly intertwined, providing the robotic system with the required knowledge for an autonomous interaction with its surrounding environment, even when it is dynamic and/or unknown. Nevertheless, this *egocentric* spatial representation provides depth information relative to the current system's position with respect to the surrounding space. In addition, the disparity estimation, and consequently the depth estimation, depends on the fixation point of the visual system since a zero disparity is obtained on that point. So, although this representational frame allows the system to act upon its environment for the purposes of locating, reaching, and/or manipulating objects, an indexed-to-a-world-based coordinate system (i.e., *allocentric*) is required for properly locating objects within a scene at any time, considerably improving the efficiency in the coordination of sensory input and motor output. Thus, the built *depth map* is abstracted to an appropriate *allocentric* representation that provides the system with the ability to reason and infer information, otherwise unachievable, necessary to autonomously act in any scenario. For this, a qualitative spatial representation together with a reasoning mechanism is used.

### 2.2. Depth Estimation: A Qualitative Approach

According to psychological studies, qualitative reasoning models provide a bridge between the perceptual and the conceptual approach by imposing discrete, symbolic frameworks on the continuous work. So, qualitative representations establish a natural connection to their quantitative representation by the abstraction of the physical world, emulating the *human* spatial cognition. Therefore, qualitative approaches provide the atomic representation to build higher-level cognitive functions by enabling any robot system to integrate perception, reasoning, and action and make predictions about spatial relations, even when precise quantitative information is not available.

In this sense, a growing trend is to obtain qualitative knowledge from images. So, for instance, Quattoni and Torralba [34] presented a prototype-based model to classify images of indoor scenes in semantic categories (e.g., bathroom and kitchen) by using a learning distance for object recognition and training on a dataset. Oliva and Torralba [35] proposed a computational model for classifying perceptual properties (e.g., naturalness, openness, roughness, expansion, and ruggedness) into semantic categories such as street, highway, or coast. Going a step further, Qayyum and Cohn [36] used more than one concept to semantically describe landing images, while Lim and Chan [37] labelled images of natural scenes by means of a fuzzy qualitative approach. On their behalf, Buoncompagni et al. [38] represented the robot environment using a fuzzy ontology.

Nevertheless, a very few approaches describe real images as a set of components arranged in the space. In fact, visual spatial information is typically encoded by a *binary* relation model of spatial entities. Furthermore, most of the calculi are based on a single aspect of space (e.g., direction, topology, distance, or position) what may be ambiguous unless a reference coordinate system is provided. In this context, one of the most well-known approaches is the region connection calculus (RCC) [39]. This method is based on extended spatial entities (regions) and the relationships between them (connections). Despite its strength in describing and reasoning about spatial structures (especially for topological structures), it is based on topological relations instead of positional information. In addition, the spatial objects are represented by regions that can be difficult to extract from a visual input when real-world scenarios are considered.

On the other hand, Freksa and Zimmermann [40] proposed an approach based on directional orientation information and motivated by considerations on how spatial information is available to humans and animals (i.e., directly through their perception). Despite including a description for static situations, it is not a unified representation for distance and orientation. As an alternative, Hernandez [41] proposed a cognitive model of space based on space nature, although it avoids falsifying the effects of an exact geometric approach. Those effects are likely due to the common limited perception acuity. As an improvement, Clementini et al. [42] developed a unified framework for qualitative representation of positional information in 2D space by combining the distance and orientation relationships. An advanced research integrates qualitative spatial reasoning with reasoning about actions and change [43–45]. Despite the very appealing idea of these approaches for reasoning in agent's control, the underlying concept of spatial neighbourhood based on the dipole calculus can result in a high time consumption, what makes it inappropriate for robot tasks. An early approach integrating qualitative representations and reasoning for positional information for domestic service robotics domains was presented in [46]. However, on the way to autonomous systems, the robotics domains cannot be constrained to domestic environments.

**Figure 2:**
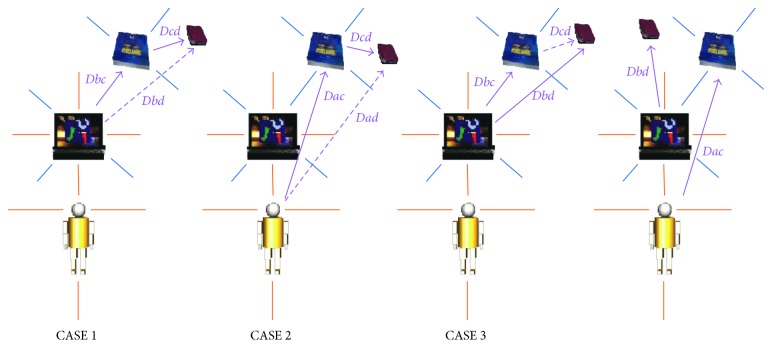
Four different atomic cases for qualitative depth estimation (integration).

So, with the aim of overcoming all these issues, we have developed a method for adequately representing and reasoning with qualitative depth based on our previous work [47]. The implemented qualitative model allows the system to properly deal with situations in which the quantitative information is not sufficiently precise, and a number of distinctions that are of interest can provide the system with the ability to properly act and interact with the environment, independently of the extension of the surrounding elements. For this, depth relations (resulting from disparity estimation) are combined to orientation. In this way, it provides a restricted form of positional information that is mainly useful in small-scale environments like objects in a room. The integration of qualitative orientation information to the qualitative distance model is especially important for the inference process [40, 48]. This integration leads to ternary relationships such that four points are required to infer new knowledge. The distance can be measured from the first point of the front/back dichotomy of the reference system (RS) or from the second point of the front/back dichotomy of the RS. Therefore, it is possible to define four different atomic cases for the reasoning process (see Figure 2 where dashed lines correspond to the distance relationship to be inferred).

- *CASE 1*: given the depth relationships *c,ab from 1st* (represented in the Figure 2 by *Dbc*) and *d,bc from 1st* (*Dcd*)—that is, both depth cues are measured from the first point of the front/back dichotomy of the RS—the relationship *d,ab from 1st* (*Dab*) is obtained
- *CASE 2*: given the depth relationships *c,ab from 2nd* (*Dac*) and *d,bc from 1st* (*Dcd*)—that is, the first depth cue is measured from the second point of the front/back dichotomy of the RS and the second depth relationship is obtained from the first point of the front/back dichotomy of the RS—the relationship *d,ab from 2nd* (*Dad*) is found
- *CASE 3*: given the relationships *c,ab from 1st* (*Dbc*) and *d,bc from 2nd* (*Dbd*)—that is, the first depth cue is measured from the first point of the front/back dichotomy of the RS, whereas the second relationship is obtained from the second point of the front/back dichotomy of the RS—the relationship *d,bc from 1st* (*Dcd*) is inferred
- The fourth case occurs when both depth cues (*Dac* and *Dbd*) are measured from the second point of the front/back dichotomy of the RS. In this case, the depth relationships are independent; therefore, it is not possible to derive any further information unless the depth relationship between the origin entities is known by means of another relationship.

As a consequence, the implemented procedure to obtain the resulting depth relationship depends on the orientation relationships. An analysis of the different orientation relationships reveals that, for *CASE 1*, the depth relationship inferred is the qualitative sum of the qualitative depths when the orientations of *Dbc* and *Dcd* are the same and the qualitative difference of the qualitative distances when these orientations are the opposite. By means of a similar reasoning process, *CASE 2* is solved. In this case, the involved depth cues are *Dbc* and *Dcd*, although the concepts of *same* and *opposite* orientations have changed. In both cases, the same and opposite orientations will determine the upper and lower bounds for the composition of heterogeneous depth ranges in any orientation. Nevertheless, for the *CASE 3*, the resulting depth relationship (*Dcd*), when the depth cues *Dbc* and *Dbd* are in the same orientation, is obtained by solving the qualitative difference between the qualitative depth cues, whereas when these depth cues are in the opposite orientation, the qualitative sum of those qualitative depth cues will be required. In this case, the same orientation will determine the lower bound of the result, and the opposite orientation will determine the upper bound when the depth cues are measured from any orientation. This knowledge has been taken into account in the implemented inference algorithm.

**Figure 3:**
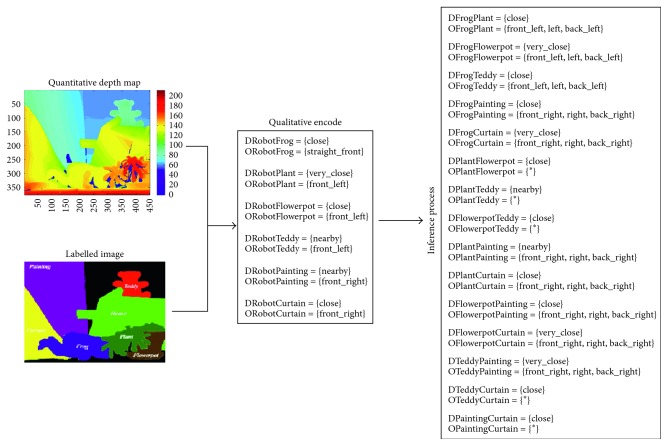
Qualitative encode for the wooden house from the estimated depth map and an image labelling all the objects of interest for the robotic system. For this, the depth reference system (DRS) has been defined as Δ_*r*_ = {[0, 60[, [60, 100[, [100, 150[, [150, 250[, [250, ∞[} and *Q*  = {very_close (vc), close (c), nearby (n), far (f), very_far (vf)}, while the relationships are coded according to the Freksa and Zimmermann's approach [48] (i.e., LAB_0_ = {front_left (fl), straight_front (sf), front_right (fr), left (l), none (n), back_left (bl), straight_back (sb), back_right (br)} and INT_0_ = {]90, 180[, [90, 90], [180, 180], _, [0, 0], ]180, 270[, [270, 270], ]270, 360[}).

Therefore, with the aim of obtaining an appropriate *allocentric* representation of the scene, the quantitative depth map must be encoded in a qualitative way. For this, in addition to the depth map, an image where the interest objects have been segmented and labelled is provided. Note that in the current version, the input images are labelled by hand although its implementation is part of our future work. So, for each target object, the object-to-object relationships are obtained from the self-to-object relationships provided by the depth map (Figure 3). In particular, in our current implementation, the depth relationships are determined by Δ_*r*_ = {[0, 60[, [60, 100[, [100, 150[, [150, 250[, [250, ∞[} and *Q* = {very_close (vc), close (c), nearby (n), far (f), very_far (vf)}, while the orientation relationships are coded according to the Freksa and Zimmermann's approach [48] (i.e., LAB_0_ = {front_left (fl), straight_front (sf), front_right (fr), left (l), none (n), right (r), back_left (bl), straight_back (sb), back_right (br)} and INT_0_ = {]90, 180[, [90, 90], ]0, 90[, [180, 180], _, [0, 0], ]180, 270[, [270, 270], ]270, 360[}). From these definitions, the qualitative relationships for each object of interest can be established and the object-to-object relationships can be inferred by means of the inference process. It is worth noting that each object of interest is represented by its centroid to properly determine the orientation relationship, whereas the orientation reference system is centred at the robot vision system.

## 3. Experimental Results

**Figure 4:**
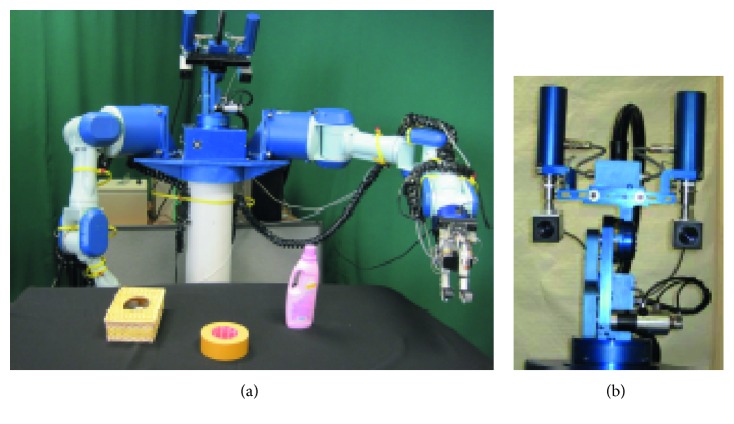
Experimental setup: external view of the used humanoid robot (a) and a detailed view of pan-tilt-vergence head (b).

As a proof of concept for the presented approach, a robotic application has been developed. For this, a humanoid torso endowed with a pan-tilt-vergence stereo head and two multijoint arms (Figure 4) was used. The head mounts two cameras separated 270 mm that can acquire colour images at 30 Hz with a resolution of 1024 × 768 pixels.

So, with the aim of evaluating the system's performance, two different kinds of experiments were carried out. On the one hand, the two constituent approaches have been analysed separately, and on the other hand, the whole performance has been examined.

### 3.1. Separate Performance Evaluation

Firstly, the performance of the different approach components has been assessed. This analysis was carried out by following the flow of the approach.

Thus, we start with the disparity estimation approach. For this, some experiments were carried out on image pairs from Middlebury dataset for Stereo Evaluation [49], which allows a quantitative comparison, thanks to the availability of the disparity ground-truth for each stereo image pair. Aimed at evaluating the accuracy in feature extraction of the disparity estimation module, we analysed and compared its performance with other band-pass representations. For this reason, the integer-based measures proposed in the dataset are not used. In its stead, the mean and standard deviation of the absolute disparity error by comparing the results with the ground-truth have been obtained. As summarised in Table 1, where average and standard deviation of the absolute disparity error expressed in pixels, three classes of filters are used for comparison: Gabor-like kernels, spherical quadrature filters (SQF), and steerable filters (second (s2) and fourth order (s4)). Note that the used parameters for estimating the disparity maps with the *PBBDE 2D shift* were 6 scales and an energy threshold of 10^−6^. The obtained average and standard deviation of the absolute disparity error, expressed in pixels, highlight that our approach has better results than Gabor filters, which are slightly better than those for the fourth-order steerable filters (s4). The second-order filters (s2), comparable with those obtained by the spherical quadrature filters (SQF), yield results about twice as bad as the fourth-order filters.

Then, the accuracy in depth estimation from the generated disparity maps was studied. For this, three different disparity situations were considered: only horizontal disparity, only vertical disparity, and both horizontal and vertical disparities exist. So, after applying (5) for depth estimation, the generated depth maps have an error less than 1 cm, making this approach suitable for robotic tasks.

Finally, the inference process is evaluated. With that purpose, two different depth reference systems (DRSs) have been defined according to two levels of granularity. Thus, the DRS for the coarse level is composed of *Q*_1_ = {close (c), nearby (n), far (f)} and Δ_*r*1_ = {[0, 40[, [40, 60[, [60, ∞[}, while for the DRS for the fine level is defined by *Q*_2_ = {closer_than_halfway (ch), halfway (h), closer_than (ct), nearby (n), closer_than_twice (ctw), double (d), further_than_double (fd) } and Δ_*r*2_ = {[0, 20[, [20, 30[, [30, 40[, [40, 60[, [60, 80[, [80, 120[, [120, ∞[}.

Thus, with the two defined depth reference systems DRS_1_ and DRS_2_, the basic step of the inference process (BSIP) for the concept of depth is solved and illustrated by means of the corresponding composition tables (Tables 2 and 3). The first column of both tables refers to the depth relationship *b* with respect to *a* (*Dab*), and the first row corresponds to the depth relationship *c* with respect to *b* (*Dbc*). The rest of cells of both tables indicate the inferred depth relationship which is included into brackets because, sometimes, it contains a disjunction of relations. Note that both tables are symmetric with respect to the main diagonal. For that reason, it is possible to represent only the upper or lower part of both tables. The values given in bold can be omitted if we only consider the upper part of these tables. These composition tables have been obtained by using the proposed algorithm, and the obtained results are the same to the handwritten ones [50].

### 3.2. Whole Approach's Performance Evaluation

**Figure 5:**
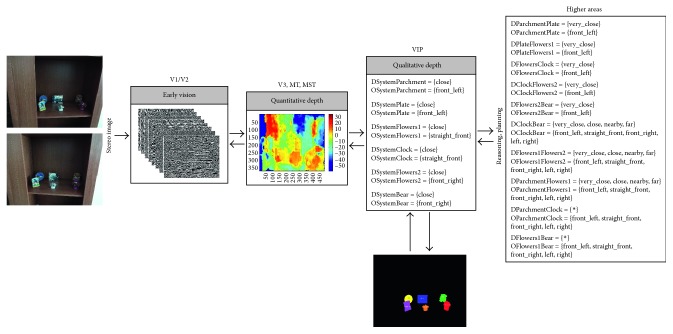
Samples of our architecture's performance when the depth reference system is defined as Δ_*r*_ = {[0, 60[, [60, 100[, [100, 120[, [120, 250[, [250, ∞[} and *Q* = {very_close (vc), close (c), nearby (n), far (f), very_far (vf)}, while the orientation relationships are coded according to the Freksa and Zimmermann's approach [48] (i.e., LAB_0_ = {front_left (fl), straight_front (sf), front_right (fr), left (l), none (n), right (r), back_left (bl), straight_back (sb), back_right (br)} and INT_0_ = {]90, 180[, [90, 90], ]0, 90[, [180, 180], _, [0, 0], ]180, 270[, [270, 270], ]270, 360[}).

**Figure 6:**
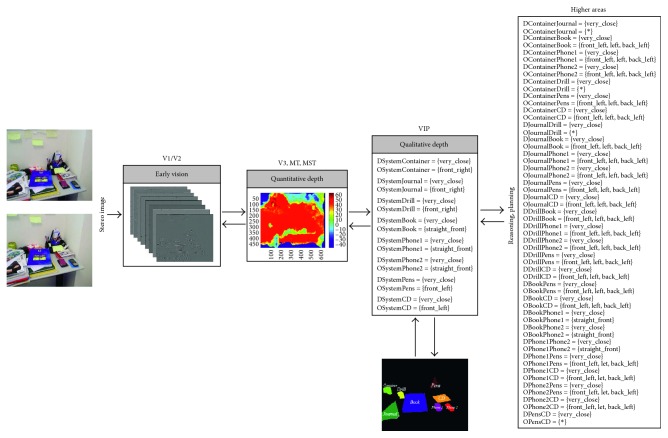
Samples of our architecture's performance when the depth reference system is defined as Δ_*r*_ = {[0, 60[, [60, 100[, [100, 120[, [120, 250[, [250, ∞[} and *Q* = {very_close (vc), close (c), nearby (n), far (f), very_far (vf)}, while the orientation relationships are coded according to the Freksa and Zimmermann's approach [48] (i.e., LAB_0_ = {front_left (fl), straight_front (sf), front_right (fr), left (l), none (n), right (r), back_left (bl), straight_back (sb), back_right (br)} and INT_0_ = {]90, 180[, [90, 90], ]0, 90[, [180, 180], _, [0, 0], ]180, 270[, [270, 270], ]270, 360[}).

**Figure 7:**
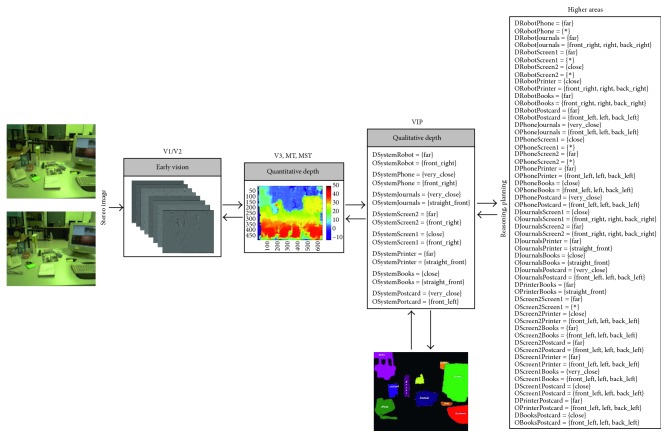
Samples of our architecture's performance when the depth reference system is defined as Δ_*r*_ = {[0, 60[, [60, 100[, [100, 120[, [120, 250[, [250, ∞[} and *Q* = {very_close (vc), close (c), nearby (n), far (f), very_far (vf)}, while the orientation relationships are coded according to the Freksa and Zimmermann's approach [48] (i.e., LAB_0_ = {front_left (fl), straight_front (sf), front_right (fr), left (l), none (n), right (r), back_left (bl), straight_back (sb), back_right (br)} and INT_0_ = {]90, 180[, [90, 90], ]0, 90[, [180, 180], _, [0, 0], ]180, 270[, [270, 270], ]270, 360[}).

**Figure 8:**
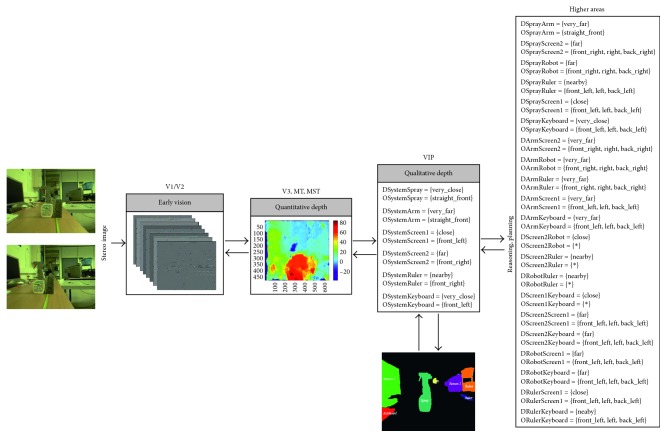
Samples of our architecture's performance when the depth reference system is defined as Δ_*r*_ = {[0, 60[, [60, 100[, [100, 120[, [120, 250[, [250, ∞[} and *Q* = {very_close (vc), close (c), nearby (n), far (f), very_far (vf)}, while the orientation relationships are coded according to the Freksa and Zimmermann's approach [48] (i.e., LAB_0_ = {front_left (fl), straight_front (sf), front_right (fr), left (l), none (n), right (r), back_left (bl), straight_back (sb), back_right (br)} and INT_0_ = {]90, 180[, [90, 90], ]0, 90[, [180, 180], _, [0, 0], ]180, 270[, [270, 270], ]270, 360[}).

**Figure 9:**
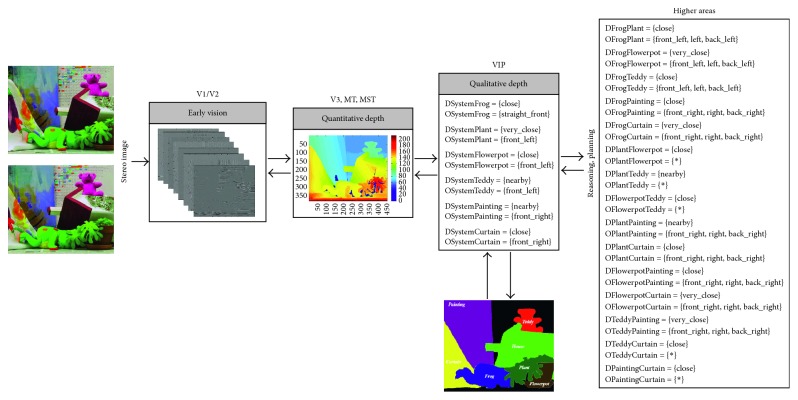
Samples of our architecture's performance when the depth reference system is defined as Δ_*r*_ = {[0, 60[, [60, 100[, [100, 120[, [120, 250[, [250, ∞[} and *Q* = {very_close (vc), close (c), nearby (n), far (f), very_far (vf)}, while the orientation relationships are coded according to the Freksa and Zimmermann's approach [48] (i.e., LAB_0_ = {front_left (fl), straight_front (sf), front_right (fr), left (l), none (n), right (r), back_left (bl), straight_back (sb), back_right (br)} and INT_0_ = {]90, 180[, [90, 90], ]0, 90[, [180, 180], _, [0, 0], ]180, 270[, [270, 270], ]270, 360[}).

Once the performance of the constituent approaches has been analysed, the whole architecture's performance is studied. So, from a stereo image (in retinoptic coordinates), a six-level image pyramid is obtained and filtered by a set of 8 oriented Gabor filters as early processing for feature extraction (Figures 5–9). From these image features, the system is able to generate a disparity map without requiring precise calibration information (in terms of the relative orientation of the cameras). This map together with the internal camera parameters results in an *egocentric* depth map since the depth cue is expressed in the camera's reference system. As shown in the examples in dark blue (Figures 5–9), no disparity and, consequently, no depth information are provided for areas monocularly visible (i.e., present in one or another stereo image but not both of them) and for textureless objects (e.g., walls). This lack of information, also common in novel sensors providing depth-like RGB-D cameras, requires an abstraction mechanism to infer the missing data and/or reduce the uncertainty.

**Figure 10:**
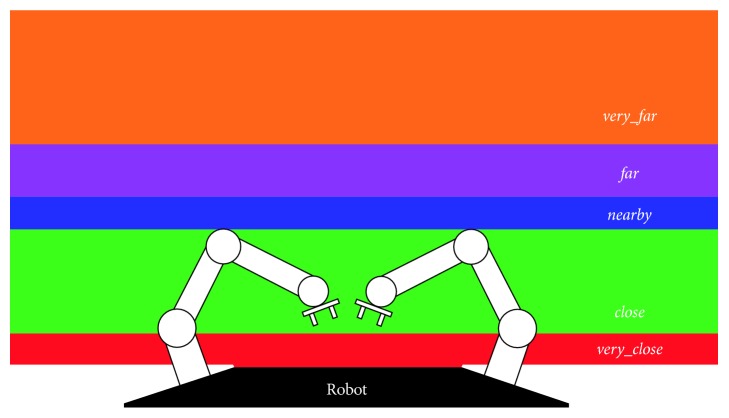
Qualitative depth areas defined according to the robot's reach.

In our case, that mechanism is the qualitative reasoning approach. For this, firstly, the depth reference should be established. With that aim, as illustrated in Figure 10, the surrounding space has been divided into five different areas based on the robot's reach. So, there is an area that cannot be reached due to its proximity to the robot's body and the arm range. This area covers from 0 cm (the robot itself) to 60 cm and, in our experiments, it has been labelled as *very_close*.

The next zone, the *close* area, corresponds to the robot peripersonal space, that is, the region (between 60 cm and 100 cm) in which the objects can be grasped and manipulated in an easy way. Approaching to the mechanic limitations, the *nearby* sector is defined. So, the objects within this area are reachable with difficulty. On the contrary, the two following qualitative areas (*far* and *very_far*) are unreachable without robot motion. The distinction between them lies in the distance necessary to be workable.

**Figure 11:**
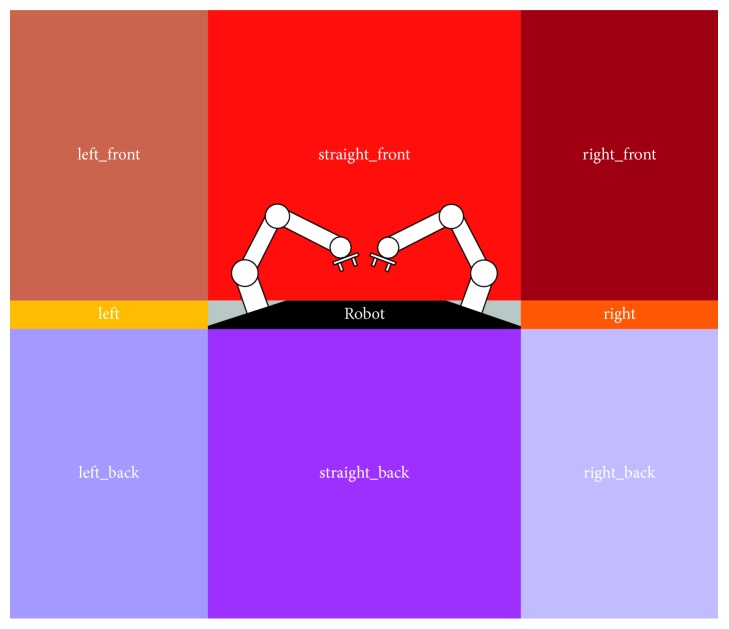
Qualitative orientations defined from the required direction information in order to properly locate and manipulate objects.

On its behalf, nine orientations have been defined based on the direction information required to properly locate (and manipulate) an object within the surrounding space (Figure 11). Note that this space division was done keeping in mind the grasping task. However, it can be adjusted to a different space distribution according to the task at hand without any cost. This is because of the qualitative approach generality.

Therefore, in our experiments, the depth reference system has been defined as Δ_*r*_ = {[0, 60[, [60, 100[, [100, 120[, [120, 250[, [250, ∞[} (expressed in cm) and *Q* = {very_close (vc), close (c), nearby (n), far (f), very_far (vf)}; while the orientation relationships are coded as LAB_0_ = {front_left (fl), straight_front (sf), front_right (fr), left (l), none (n), right (r), back_left (bl), straight_back (sb), back_right (br)} and INT_0_ = {]90, 180[, [90, 90], ]0, 90[, [180, 180], _, [0, 0], ]180, 270[, [270, 270], ]270, 360[}.

From this definition, the *egocentric* quantitative map is translated into an *egocentric* qualitative depth representation. Realise that the qualitative depth relationships from the system to the considered objects of interest can easily be obtained when the interest objects are defined by their centroid. As depicted in Figures 5–9, only some scene objects have been considered as interesting for the system (and used for generating the *egocentric* qualitative depth representation). Although in the current implementation the objects of interest have been manually segmented and labelled, it is intended to be automatically done in the future.

**Figure 12:**
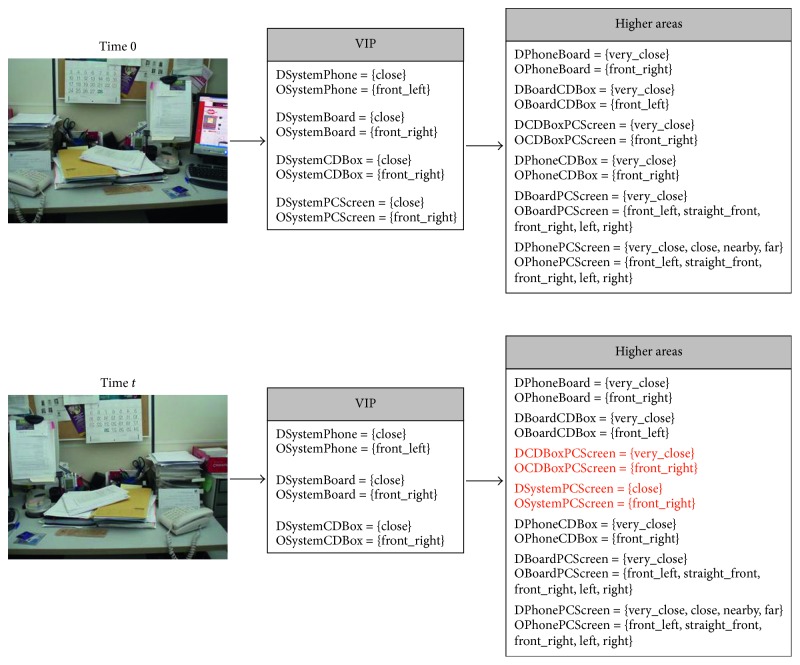
An example of the *allocentric* contribution for performing robot tasks. A visual scrutiny of the surrounding space is performed along time. While the PC screen is visible (and detected) at time 0, it is missed at time (*t*). However, the *allocentric* depth data provide the robot with the necessary information to accurately plan the robot arm path to grasp the CD box without colliding with the PC screen (red relationships).

The *egocentric* qualitative scene representation is the input for the higher areas where the inference process takes place. In this way, an *allocentric* representation (i.e., object-to-object) is obtained (Figures 5–9). It is worth noting that the inference process is applied for each new stereo image and is repeated until no more information can be obtained. In this way, depth data independent of the robot's current location are generated. This is a key issue since these depth data survive over extended periods of time providing the robot with a conscious perception of the objects around it. That is, the robot is endowed with a *cognitive* representation of the whole visual surrounding space, what is essential to properly plan and execute the next robot action. A clear example is illustrated in Figure 12 (only one image of the stereo pair for simplicity), where a visual scrutiny of the surrounding space has led to change in the initial robot position. Nevertheless, thanks to the *allocentric* qualitative depth information, the robot knows that a PC screen is located very close to the CD box and at the right side of it despite its invisibility. In this way, a success path free of obstacles can be properly generated.

On the contrary, the *egocentric* representations are temporary, relying on immediate visual input. So, this robot-to-object depth information depends on the current robot position, and it can be used to focus the robot's attention on a particular zone of the perceptive field, that is, on the object to work with. As a consequence, the combination of both depth representations is required to acquire a natural behaviour flexible enough to successfully perform robot tasks.

## 4. Conclusions

With the purpose of providing robot systems with the capabilities of integrating reasoning, perception, and action, an analysis of the human visual cortex is the starting point. In this sense, neuroscientific studies have shown that it is hierarchically organised progressively abstracting away from the particularities at the bottom processing levels. So, visual depth information must be initially coded in retinotopic space, while that information must be ultimately coded in head-centred and/or body-centred representations. Aiming at emulating that behaviour, we presented a biologically inspired multilevel architecture for robot depth estimation, which combines different methods into a coherent whole. Thus, based on the dorsal *where* pathway design, from a stereo image pair, the early vision (V1/V2-level) is modelled as a set of complex Gabor filters with a cosine-based real part and a sine-based imaginary part. This processing at multiple scales and orientations results in a quantitative stereo disparity estimation. This estimation allows the system to easily compute other features such as depth, optic flow, or orientation, from the filtering stage. In addition, the correspondence estimation and autocalibration are performed simultaneously by the proposed approach, not requiring precise calibration information (in terms of the relative orientation of the cameras).

From this knowledge, an *egocentric*, abstract representation of the scene object localization is obtained by following a qualitative approach since the ability to reason in and about space is crucial for a robotic system involved in physical actions and decisions. Finally, a qualitative reasoning method allows the system to infer new information (otherwise unavailable) and make decisions more accurately. Actually, that qualitative representation connects the low-level quantitative data with their corresponding higher-level cognitive functions. This connection is required for the perception-action cycle present in intelligent Robotics. In addition, it helps to overcome some quantitative deficiencies as the missing information when textureless areas are observed.

The approach's performance has been evaluated in several scenarios. The results show that it is able to properly deal with uncertainty by means of the combination of the visual processes despite the difficulty in combining different biological vision algorithms. However, this system is focused on depth estimation. Not considering other cues results in a lack of generality and robustness inherent in the primate visual system. Thus, there are still many challenges to be solved on the way to achieve a complete biological active vision system. Consequently, our future work is aimed at broadening the information extracted from a visual input as well as working in the integration with other sensory data to gather more robust information about the environment to interact with.

## Figures and Tables

**Table 1:** Quantitative comparison (average and standard deviation of the absolute errors in pixels) in disparity estimation between different band-pass representations on Middlebury images [49].

	Venus		Sawtooth		Tsukuba	
	Avg.	Std. dev.	Avg.	Std. dev.	Avg.	Std. dev.
Gabor	0.25	0.77	0.41	1.26	0.32	0.61
s4	0.40	1.30	0.50	1.86	0.36	0.68
s2	0.98	2.44	1.12	2.50	0.47	0.79
SQF	0.95	2.40	0.93	2.20	0.46	0.85
*PBBDE 2D shift*	0.04	0.74	0.57	1.08	0.12	0.67

**Table 2:** The composition table which solves the BSIP for the coarse DRS_1_ with *Q*_1_ = {close (c), nearby (n), far (f)} such that the first column represents the relation *b* with respect to *a*, while the first row indicates the relation *c* with respect to *a*, which is included into brackets because, sometimes, it contains a disjunction (list) of relationships. The values given in bold can be omitted since table is symmetric with respect to the diagonal.

*Dab/Dbc*	c	n	f
*CASE 1*			
c	{c, n, f}	{c, n, f}	{f}
n	**{c, n, f}**	{c, n, f}	{f}
f	**{f}**	**{f}**	{c, n, f}

*CASE 2 and 3*			
c	{c}	{n}	{f}
n	**{n}**	{c}	{f}
f	**{f}**	**{f}**	{f}

**Table 3:** The composition table which solves the BSIP for the fine DRS_2_ with *Q*_2_ = {closer_than_halfway (ch), halfway (h), closer_than (ct), nearby (n), closer_than_twice (ctw), double (d), further_than_double (fd)} where the first column represents the relation *b* with respect to *a*, while the first row indicates the relationship *c* with respect to *b*. The rest of cells of the table correspond to the inferred distance relationship *c* with respect to *a*, which is included into brackets because, sometimes, it contains a disjunction (list) of relationships. The values given in bold can be omitted since table is symmetric with respect to the diagonal.

*Dab/Dbc*	ch	h	ct	n	ctw	d	fd
*CASE 1*							
ch	{ch, h, ct, n}	{ch, h, ct, n}	{ch, h, ct, n, ctw}	{h, ct, n, ctw, d}	{h, ctw, d}	{ctw, d, fd}	{fd}
h	**{ch, h, ct, n}**	{ch, h, ct, n, ctw}	{ch, h, ct, n, ctw}	{ch, h, ct, n, ctw, d}	{ct, n, ctw, d}	{n, ctw, d, fd}	{fd}
ct	**{ch, h, ct, n, ctw}**	**{ch, h, ct, h, ctw}**	{ch, h, ct, n, ctw, d}	{ch, h, ct, n, ctw, d}	{h, ct, n, ctw, d, fd}	{n, ctw, d, fd}	{fd}
n	**{h, ct, n, ctw, d}**	**{ch, h, ct, n, ctw, d}**	**{ch, h, ct, n, ctw, d}**	{ch, h, ct, n, ctw, d, fd}	{ch, h, ct, n, ctw, d, fd}	{h, ct, n, ctw, d, fd}	{fd}
ctw	**{h, ctw, d}**	**{ct, n, ctw, d}**	**{ch, ct, n, ctw, d, fd}**	**{ch, h, ct, n, ctw, d, fd}**	{ch, h, ct, n, ctw, d, fd}	{ch, h, ct, n, ctw, d, fd}	{fd}
d	**{ctw, d, fd}**	**{n, ctw, d, fd}**	**{n, ctw, d, fd}**	**{h, ct, n, ctw, d, fd}**	**{ch, h, ct, n, ctw, d, fd}**	{ch, h, ct, n, ctw, d, fd}	{fd}
fd	**{fd}**	**{fd}**	**{fd}**	**{fd}**	**{fd}**	**{fd}**	{ch, h, ct, n, ctw, d, fd}

*CASE 2* and *3*							
ch	{ch}	{h}	{ct}	{n}	{ctw}	{d}	{fd}
h	**{h}**	{ch}	{ch}	{ct}	{n}	{d}	{fd}
ct	**{ct}**	**{ch}**	{ch}	{h}	{n}	{d}	{fd}
n	**{n}**	**{ct}**	**{h}**	{ch}	{ct}	{ctw}	{fd}
ctw	**{ctw}**	**{n}**	**{n}**	**{ct}**	{ch}	{n}	{fd}
d	**{d}**	**{d}**	**{d}**	**{ctw}**	**{n}**	{ct}	{fd}
fd	**{fd}**	**{fd}**	**{fd}**	**{fd}**	**{fd}**	**{fd}**	{fd}

## Data Availability

The data used to support the findings of this study are available from the corresponding author upon request.
